# Notes on the Genera *Graphidessa* Bates, 1884, *Pararondibilis* Breuning, 1961, *Paratimiola* Breuning, 1965, and *Tuberenes* Breuning, 1978 (Coleoptera, Cerambycidae, Lamiinae)

**DOI:** 10.3390/insects16050488

**Published:** 2025-05-02

**Authors:** Guiqiang Huang, Yechen Sun, Shaofu Ji, Chengyuan Su

**Affiliations:** School of Biological Science and Technology, Liupanshui Normal University, Liupanshui 553004, China; hgqnasa@lpssy.edu.cn (G.H.);

**Keywords:** taxonomy, replacement name, checklist, nomen nudum

## Abstract

Taxonomic reviews of some species of *Graphidessa* Bates, 1884, *Pararondibilis* Breuning, 1961, *Paratimiola* Breuning, 1965, and *Tuberenes* Breuning, 1978 are provided herein. The following new combinations are proposed: *Graphidessa variegata* Hayashi, 1974, *Tuberenes minuta* (Pic, 1925), and *Tuberenes sikkimensis* Breuning, 1978 are transferred to *Pararondibilis*; *Tuberenes vietnamensis* Breuning, 1972 is transferred to *Paratimiola*. The replacement name *Pararondibilis rubina* **nom. nov**. is proposed for *Pararondibilis sikkimensis* (Breuning, 1978). Updated species checklists for *Graphidessa*, *Pararondibilis*, *Paratimiola*, and *Tuberenes* are provided. Keys to species of *Graphidessa* and *Pararondibilis* are provided. *Tuberoenes* Breuning, 1972 is a nomen nudum of *Tuberenes*.

## Simple Summary

The genera *Graphidessa* Bates, 1884 (presently consisting of four species and one subspecies), *Pararondibilis* Breuning, 1961 (presently consisting of five species), *Paratimiola* Breuning, 1965 (presently consisting of one species), and *Tuberenes* Breuning, 1978 (presently consisting of four species) are small groups of the subfamily Lamiinae. The genera *Graphidessa* and *Tuberenes* have been studied well, but we found that some species of it should be transferred to the genera *Pararondibilis* and *Paratimiola*. In this paper, we provide a taxonomic review of some species of these four genera.

## 1. Introduction

Bates established *Graphidessa* for *Graphidessa venata* Bates, 1884 [1], and Liu et al. described *Graphidessa jinfoensis* (from China) and made a key to all species [2]. *Graphidessa* presently consists of four species and one subspecies from East Asia [3]. Breuning established *Pararondibilis* for *Pararondibilis sikkimensis* Breuning, 1961 (from India) [4]; subsequently, *Pararondibilis acrosa* Holzschuh, 2003 (from Nepal), *P*. *eluta* Holzschuh, 2003 (from Nepal), *P*. *macularia* Holzschuh, 2003 (from India), and *Pararondibilis pinicola* Holzschuh, 2018 (from Nepal) were described [5,6]. *Pararondibilis* presently consists of these five species [3]. Breuning established *Paratimiola* for *Paratimiola rondoni* Breuning, 1965 (from Laos) [7], which is presently the only described species of the genus [3]. Breuning established *Tuberenes* for *Eryssamena robustipes* Pic, 1939 (from China); he also described *Tuberenes sikkimensis* (from India) and transferred *Eryssamena minuta* Pic, 1925 (from Vietnam) and *Tuberoenes vietnamensis* Breuning, 1972 (from Vietnam) to *Tuberenes* [8]. *Tuberenes* presently consists of these four species [3].

We found some issues suggesting that the taxonomic status of *Graphidessa variegata* Hayashi, 1974, *Tuberenes minuta* (Pic, 1925), *Tuberenes sikkimensis* Breuning, 1978, and *Tuberenes vietnamensis* Breuning, 1972 are doubtful. Therefore, this paper reviews these issues.

## 2. Materials and Methods

The specimens examined in this study are deposited in the following institutional and private collections:

**BPBM** Bernice Pauahi Bishop Museum, Honolulu, HI, USA;

**CHS** Collection Carolus Holzschuh, Villach, Austria;

**HNHM** Hungarian Natural History Museum, Budapest, Hungary;

**IZCAS** Insect collection of the Institute of Zoology, Chinese Academy of Sciences, Beijing, China;

**MNHN** Muséum National d’Histoire Naturelle, Paris, France.

The copyrights of other photographs were added to the legends of the corresponding figures. All photographs and figures herein were produced using Photoshop CS5 software (Adobe company, San Jose, CA, USA).

## 3. Results

***Pararondibilis* Breuning, 1961**

*Pararondibilis* Breuning, 1961: 547 [4]; Breuning 1963: 523 (catalogue) [9]; Löbl and Smetana 2010: 210 (catalogue) [10]; Lin 2015: 158 (catalogue) [11]; Kariyanna et al., 2017: 92 (catalogue) [12]; Danilevsky 2020: 296 (catalogue) [13]. Type species: *Pararondibilis sikkimensis* Breuning, 1961.

Comparative analyses of the holotypes of *Graphidessa variegata* Hayashi, 1974, *Tuberenes minuta* (Pic, 1925), and *Tuberenes sikkimensis* Breuning, 1978 reveal that these species do not belong to *Graphidessa* or *Tuberenes* because they lack several diagnostic characteristics of these genera: the post-basal bump on each elytron is not covered with a small fascicle of setae; each elytron is not covered with short white or pale yellow pubescence forming several longitudinal lines at the apical half; and the femora is not covered with erect pubescence.

**Figure 2:**
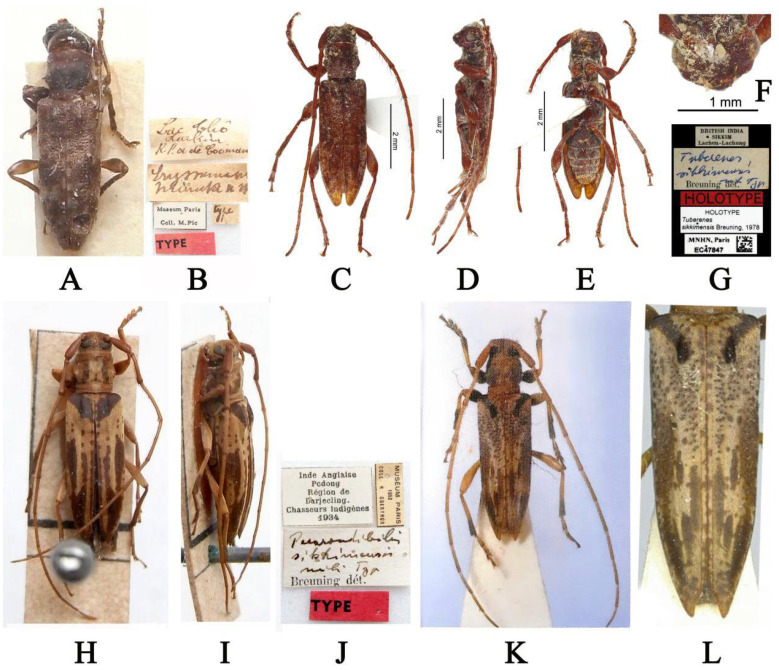
(**A**,**B**) *Eryssamena minuta*, holotype: (**A**) dorsal habitus; (**B**) labels (photographs (**A**,**B**) were taken by Xavier Gouverneur). (**C**–**G**) *Tuberenes sikkimensis*, holotype, male: (**C**) dorsal habitus; (**D**) lateral habitus; (**E**) ventral habitus; (**F**) frontal habitus; (**G**) labels (photographs (**C**–**G**) were taken by Christophe Rivier). (**H**–**J**) *Pararondibilis sikkimensis*, holotype: (**H**) dorsal habitus; (**I**) lateral habitus; **J** labels (photographs (**H**–**J**) were taken by Xavier Gouverneur). (**K**,**L**) *Graphidessa variegata*, holotype, male: (**K**) dorsal habitus (photograph (**K**) was reproduced from Mizuno and Shiyake 2004 [24]); (**L**) elytra (photograph (**L**) was reproduced from Liu et al., 2023 [2]).

However, comparative analyses of the holotypes of *Pararondibilis sikkimensis* (Figure 2H,I), *Eryssamena minuta* (now *Tuberenes minuta*) (Figure 2A), *Tuberenes sikkimensis* (Figure 2C–F), and *Graphidessa variegata* (Figure 2K,L) reveal that the latter three species belong to the genus *Pararondibilis* based on the shared commonality of morphological characteristics with *P*. *sikkimensis*: the scape is short and quite thick, antennomere III is longer than the scape, antennomere III is shorter than antennomere IV, the pronotum has a tubercle at the middle sides, each elytron has a post-basal bump, and the mesocoxal cavities open externally to the mesepimera.

To date, *Pararondibilis* consists of eight species.

**Distribution:** China, India, Nepal, Vietnam.

***Pararondibilis acrosa* Holzschuh, 2003**

*Pararondibilis acrosa* Holzschuh, 2003: 313 (type locality: “Nawakot, Trisuli Khola, Dhunche-Syabru Bensi, Nepal”), pl. VI, fig. 11 (holotype, male) [5]; Weigel 2006: 506 (catalogue) [14]; Löbl and Smetana 2010: 210 (catalogue) [10]; Lin 2015: 158, figs. page 158 bas and 159 (paratypes) [11]; Danilevsky 2020: 296 (catalogue) [13].

**Figure 1:**
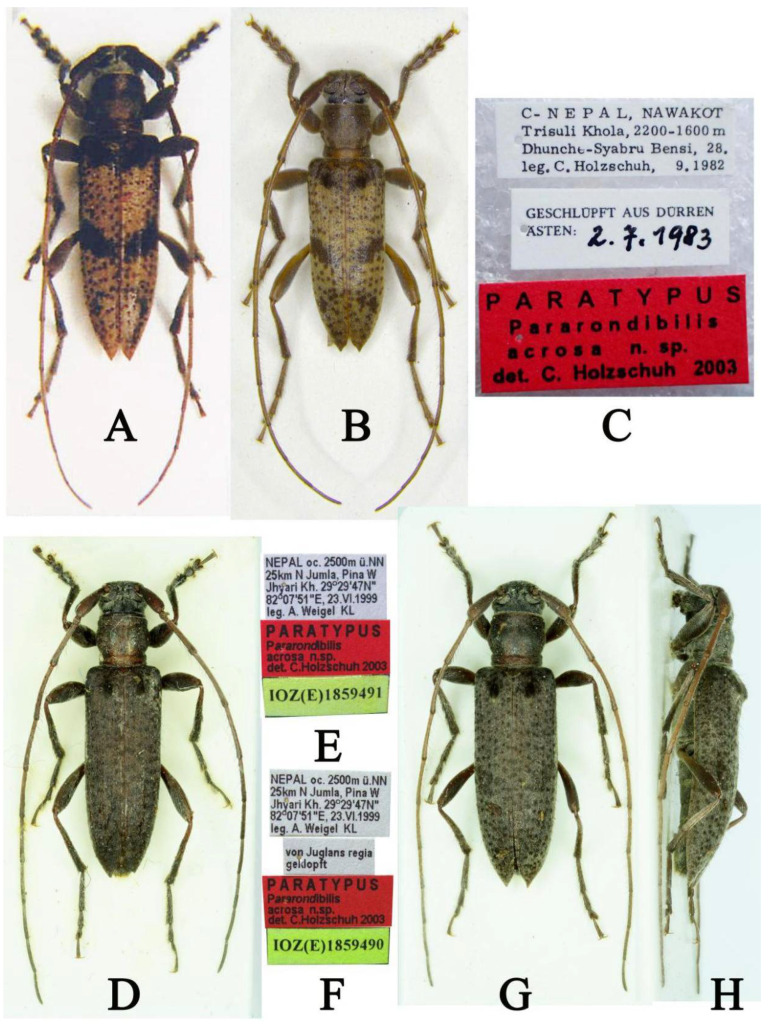
*Pararondibilis acrosa* types: (**A**) Holotype, male, dorsal habitus (photograph (**A**) was reproduced from Holzschuh 2003 [5]). (**B**–**H**) Paratypes: (**B**) dorsal habitus; (**C**) labels (photographs (**B**,**C**) were taken by Andreas Weigel); (**D**) male, dorsal habitus; (**E**) labels; (**F**) labels; (**G**) male, dorsal habitus; (**H**) male, lateral habitus (photographs (**D**–**H**) were taken by Mei-Ying Lin).

**Type material examined:** One paratype (CHS), C-NEPAL, NAWAKOT, Trisuli Khola, 2200–1600 m Dhunche-Syabru Bensi, 28.IX.1982, leg. C. Holzschuh (printed with black ink on a rectangular white label)/GESCHLÜPFT AUS DÜRREM ÄSTEN: 2.7.1983 (“GESCHLÜPFT AUS DÜRREM ÄSTEN:” printed and “2.7.1983” handwritten with black ink on a rectangular white label)/PARATYPUS Pararondibilis acrosa n. sp. det. C. Holzschuh 2003 (printed with black ink on a rectangular red label), examined from two photographs (Figure 1B,C); one paratype, ♂ (IZCAS), NEPAL oc. 2500 m ü.NN 25 km N Jumla, Pina W Jhyari Kh. 29°29′47N″ 82°07′51″E, 23.VI.1999 leg. A. Weigel KL (printed with black ink on a rectangular white label)/PARATYPUS *Pararondibilis acrosa* n.sp. det.C. Holzschuh 2003 (printed with black ink on a rectangular red label)/IOZ(E)1859491 (printed with black ink on a rectangular yellow label), examined from two photographs (Figure 1D,E); one paratype, ♂ (IZCAS), NEPAL oc. 2500 m ü.NN 25km N Jumla, Pina W Jhyari Kh. 29°29′47N″ 82°07′51″E, 23.VI.1999 leg. A. Weigel KL (printed with black ink on a rectangular white label)/von Juglans regia geldopft (printed with black ink on a rectangular white label)/PARATYPUS *Pararondibilis acrosa* n.sp. det. C.Holzschuh 2003 (printed with black ink on a rectangular red label)/IOZ(E)1859490 (printed with black ink on a rectangular yellow label), examined from three photographs (Figure 1F–H).

**Distribution:** Nepal (Dhunche-Syabru Bensi).

**Remarks:** Holzschuh described the body color of this species as variable from reddish-brown to blackish, with elytra either without or with two dark transverse bands (a narrow one extending between the humeri and covering the post-basal bumps, another one ascending somewhat obliquely to the lateral margin in the center, usually not touching either the lateral margin or the suture) [5]. However, we found the holotype (Figure 1A) and two paratypes (Figure 1D,G,H) have very different colors of their antennae, pronotum, elytra, and leg, except for the characteristics mentioned above. Therefore, we suggest that the two paratypes may not be *Pararondibilis acrosa*. Andreas Weigel told the senior author that Carolus Holzschuh also thought there may be two species in the type series of *Pararondibilis acrosa*.

***Pararondibilis eluta* Holzschuh, 2003**

*Pararondibilis eluta* Holzschuh, 2003: 314 (type locality: “Dhawalagiri, Kali-Gandaki-Khola, Kalopani, Nepal”) [5]; Weigel 2006: 506 (catalogue) [14]; Löbl and Smetana 2010: 210 (catalogue) [10]; Danilevsky 2020: 296 (catalogue) [13].

**Distribution:** Nepal (Kalopani).

***Pararondibilis macularia* Holzschuh, 2003**

*Pararondibilis macularia* Holzschuh, 2003: 314 [type locality: “Jammu, Yourdu, Jammu and Kashmir (Kishtwar District), India”] [5]; Löbl and Smetana 2010: 210 (catalogue) [10]; Kariyanna et al., 2017: 92 (catalogue) [12]; Danilevsky 2020: 296 (catalogue) [13].

**Distribution:** India (Kishtwar).

***Pararondibilis minuta* (Pic, 1925) comb. nov.**

*Eryssamena minuta* Pic, 1925: 31 (type locality: “Tonkin, Vietnam”) [15]; Breuning 1963: 523 (catalogue) [9].

*Tuberenes minuta*: Breuning 1978: 21 (redescription) [8].

**Type material examined:** Holotype (MNHN): Lac Thô Tonkin R.P. a de Cooman (handwritten with black ink on a rectangular white label)/*Eryssamena minuta* n sp (handwritten with black ink on a rectangular white label)/Museum Pairs Coll. M. Pic (printed with black ink on a rectangular white label with black borders)/Type (handwritten with black ink on a rectangular white label)/TYPE (printed with black ink on a red rectangular label), examined from two photographs (Figure 2A,B).

**Distribution:** Vietnam (Tonkin).

***Pararondibilis pinicola* Holzschuh, 2018**

*Pararondibilis pinicola* Holzschuh, 2018: 504 (type locality: “Kathmandu, Nepal”), figs. 2 (holotype male), 2 (paratype female) [6]; Danilevsky 2020: 296 (catalogue) [13].

**Distribution:** India (Kumaon Himalaya), Nepal (Kathmandu).

***Pararondibilis rubina* nom. nov.**

*Tuberenes sikkimensis* Breuning, 1978: 21 (type locality: “Lachen-Lachung, Sikkim, India”) [8]; Breuning 1982: 23 (redescription) [16]; Löbl and Smetana 2010: 213 (catalogue) [10]; Danilevsky 2020: 299 (catalogue) [13].

**Type material examined:** Holotype (MNHN): BRITISH INDIA SIKKIM Lachen-Lachung (printed with black ink on a rectangular white label)/*Tuberenes sikkimensis* mihi Typ Breuning dét. (“*Tuberenes sikkimensis* mihi Typ” handwritten with blue ink and “Breuning dét.” printed with black ink on a rectangular white label)/HOLOTYPE (printed with black ink on a rectangular red label)/HOLOTYPE *Tuberenes sikkimensis* Breuning, 1978 (printed with black ink on a rectangular white label)/MNHN, Paris EC47847 plus a QR code (printed with black ink on a rectangular white label), examined from five photographs (Figure 2C–G).

**Etymology:** The new specific epithet of this species is derived from the Latin word “rubina”, referring to its dark red body.

**Distribution:** India (Sikkim).

**Remarks:** Breuning first described *Tuberenes sikkimensis* [8]; then, Breuning described *Tuberenes sikkimensis* again as a new species [16]. Therefore, the available published date of *Tuberenes sikkimensis* is 1978, following Article 23.1 of ICZN [17].

Since *T*. *sikkimensis* belongs to *Pararondibilis*, it becomes a secondary homonym of *Pararondibilis sikkimensis* Breuning, 1961. According to ICZN Art. 57.3.1, Art. 59.1, and Art. 60.3 [17], we hereby propose the replacement name *Pararondibilis rubina* **nom. nov.** for *Pararondibilis sikkimensis* (Breuning, 1978).

***Pararondibilis sikkimensis* Breuning, 1961**

*Pararondibilis sikkimensis* Breuning, 1961: 547 (type locality: “Pedong, Sikkim, India”) [4]; Breuning, 1963: 523 (catalogue) [9]; Breuning, 1977: 137 (redescription), pl. II, fig. 5 [18]; Löbl and Smetana 2010: 210 (catalogue) [10]; Kariyanna et al., 2017: 92 (catalogue) [12]; Danilevsky 2020: 296 (catalogue) [13].

**Type material examined:** Holotype (MNHN): Inde Anglaise Pedong Région de Darjecling. Chasseurs indigènes 1934 (printed with black ink on a rectangular white label with black borders)/MUSÉUM PARIS 1952 COLL R OBERTHUR (printed with black ink on a rectangular white label with black borders)/*Pararondibilis sikkimensis* mihi Typ Breuning dét (“*Pararondibilis sikkimensis* mihi Typ” handwritten and “Breuning dét” printed with black ink on a rectangular white label)/TYPE (printed with black ink on a rectangular red label), examined from three photographs (Figure 2H–J).

**Distribution:** India (Sikkim).

***Pararondibilis variegata*** **(Hayashi, 1974) comb. nov.**

*Graphidessa variegata* Hayashi, 1974: 50 (type locality: “Lishan in Tachiachi, Taichung Hsien, Taiwan, China”) [19]; Hua 1982: 88 (catalogue) [20]; Nakamura et al., 1992: 92 (catalogue) [21]; Hua 2002: 211 (catalogue) [22]; Chou 2004: 321, fig. male (misidentified) [23]; Mizuno and Shiyake 2004: 52 (catalogue), pl. 21, fig. 476 (holotype) [24]; Hua et al., 2009: 84, pl. LXXXIV, fig. 972 (male), 219 (redescription in Chinese), 361 (redescription in English) [25]; Löbl and Smetana 2010: 223 (catalogue) [10]; Nakamura et al., 2014: 155 (catalogue) [26]; Lin and Yang 2019: 260 (catalogue) [27]; Danilevsky 2020: 314 (catalogue) [13]; Liu et al., 2023: 17, fig. 1 (distribution map), 22, fig. 7C (elytra of holotype, male), 23 (key) [2].

**Figure 3:**
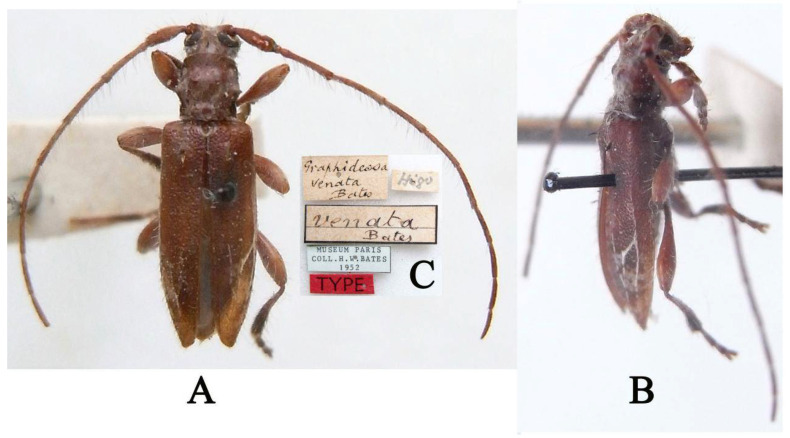
*Graphidessa venata*, holotype: (**A**) dorsal habitus; (**B**) lateral habitus; (**C**) labels (photographs (**A**–**C**) were taken by Xavier Gouverneur).

Hayashi compared *Graphidessa variegata* with two known species (*Graphidessa venata* Bates, 1884 and *Graphidessa obliquefasciata* Komiya and Kusama, 1974) of *Graphidessa* when he described it [19]. However, comparative analyses of the holotypes of *Graphidessa venata venata* Bates, 1884 (Figure 3A–C, type species of *Graphidessa*) and *G*. *variegata* (Figure 2K,L) reveal that *G*. *variegata* does not belong to *Graphidessa* and should be transferred to *Pararondibilis* Breuning, 1961, as mentioned above.

**Distribution:** China (Taiwan).

**Key to species of *Pararondibilis***

1. Elytral apices subtruncate..................................................................................................................................................................................................................***P*. *pinicola***
- Elytral apices sub-rounded or acute....................................................................................................................................................................................................................2
2. Elytral apices sub-rounded.................................................................................................................................................................................................................................3
- Elytral apices acute................................................................................................................................................................................................................................................4
3. Each elytron with a median grayish-yellow transverse band running up towards the lateral edge...........................................................................................***P*. *minuta***
- Each elytron without a median grayish-yellow transverse band running up towards the lateral edge........................................................................................***P*. *rubina***
4. Elytron without a band basally...........................................................................................................................................................................................................................5
- Elytron with a band basally..................................................................................................................................................................................................................................6
5. Elytra densely covered with short yellowish-gray pubescence..................................................................................................................................................***P*. *macularia***
- Elytra densely covered with short gray pubescence............................................................................................................................................................................***P*. *acrosa***
6. Elytra with a pair of oblique black bands in middle..........................................................................................................................................................................***P*. *acrosa***
- Elytra without a pair of oblique black bands in middle....................................................................................................................................................................................7
7. Post-basal bump on elytron angularly processed in profile.........................................................................................................................................................***P***. ***variegata***
- Post-basal bump on elytron flat in profile...........................................................................................................................................................................................................8
8. Elytra not covered with protruding pubescence........................................................................................................................................................................***P*. *sikkimensis***
- Elytra covered with protruding pubescence............................................................................................................................................................................................***P*. *eluta***

***Graphidessa* Bates, 1884**

*Graphidessa* Bates, 1884: 248 [1]; Aurivillius 1922: 231 [28]; Breuning 1963: 492 (catalogue) [9]; Breuning 1975: 7 (key) [29]; Breuning 1976: 75 (redescription) [30]; Nakamura et al., 1992: 92 (catalogue) [21]; Ohbayashi and Niisato 2007: 618 (catalogue) [31]; Löbl and Smetana 2010: 223 (catalogue) [10]; Nakamura et al., 2014: 155 (catalogue) [26]; Lin and Yang 2019: 260 (catalogue) [27]; Danilevsky 2020: 314 (catalogue) [13]. Type species: *Graphidessa venata* Bates, 1884.

As mentioned above, *Graphidessa variegata* Hayashi, 1974 was transferred to *Pararondibilis*. *Graphidessa* now consists of three species and one subspecies.

**Distribution:** China, Japan.

***Graphidessa jinfoensis* Liu et al., 2023**

*Graphidessa jinfoensis* Liu et al., 2023: 17 (type locality: “Jinfoshan National Nature Reserve, Chongqing, China; Dongfeng Lake National Wetland Park, Xishui County, Zunyi City, Guizhou Province, China”), fig. 1 (distribution map), fig. 2A (holotype, male), 2B (paratype, female), 3A (holotype, male), 3B (paratype, female), 4A (details of pronotum of holotype, male), 4B (details of pronotum of paratype, female), 4C (details of bump near base of elytra of holotype, male), 4D (details of bump near base of elytra of paratype, female), 5A–F (holotype, male genitalia), 6A–C (paratype, female ovipositor), 7D (elytra of holotype, male), 23 (key) [2].

**Distribution:** China (Chongqing, Guizhou).

***Graphidessa obliquefasciata* Komiya and Kusama, 1974**

*Graphidessa obliquefasciata* Komiya and Kusama, 1974: 138 (type locality: “Sungkang, Nantou Hsien, Taiwan, China”), pl. I [32]; Hua 1982: 88 (catalogue) [20]; Nakamura et al., 1992: 92 (catalogue) [21]; Hua 2002: 211 (catalogue) [22]; Chou 2004: 321, figs. male and female [23]; Löbl and Smetana 2010: 223 (catalogue) [10]; Nakamura et al., 2014: 155 (catalogue) [26]; Lin and Yang 2019: 260 (catalogue) [27]; Danilevsky 2020: 314 (catalogue) [13]; Liu et al., 2023: 23 (key) [2].

**Distribution:** China (Taiwan).

***Graphidessa venata venata*** **Bates, 1884**

*Graphidessa venata* Bates, 1884: 248 (type locality: “Higo, Kyushsu, Japan”) [1]; Aurivillius 1922: 321 (catalogue) [28]; Breuning 1963: 492 (catalogue) [9]; Breuning 1976: 75 (redescription) [30].

*Graphidessa venata venata* Ohbayashi and Niisato 2007: 618, pl. 67, fig. 6 (male) [31]; Löbl and Smetana 2010: 223 (catalogue) [10]; Danilevsky 2020: 314 (catalogue) [13]; Liu et al., 2023: 17, fig. 1 (distribution map), 22, fig. 7A (elytra of holotype, male), 23 (key) [2].

**Type material examined:** Holotype (MNHN): *Graphidessa venata* Bates (handwritten with black ink on a rectangular white label)/Higo (handwritten with black pencil on a rectangular white label)/*venata* Bates (handwritten with black ink on a rectangular white label with black borders and a transversal black line in middle)/MUSEUM PARIS COLL. H. W. BATES 1952 (printed with black ink on a rectangular white label with black borders)/TYPE (printed with black ink on a rectangular red label), examined from three photographs (Figure 3A–C).

**Distribution:** Japan (Kyushsu).

***Graphidessa venata takakuwai* Fujita, 1980**

*Graphidessa venata takakuwai* Fujita, 1980: 15 (type locality: “Mikurajima Island, Izu Islands, Japan”), figs. 36 and 37 [33]; Ohbayashi and Niisato 2007: 618, pl. 67, fig. 7 (male) [31]; Löbl and Smetana 2010: 223 (catalogue) [10]; Danilevsky 2020: 314 (catalogue) [13]; Liu et al., 2023: 17, fig. 1 (distribution map), 22, fig. 7B (elytra of holotype, male), 23 (key) [2].

**Distribution:** Japan (Izu Islands).

**Key to species of *Graphidessa***

1. Body dark reddish-brown or light red....................................................................................................................................................................................................................2
- Body dark brown.........................................................................................................................................................................................................................................................3
2. Elytra sparsely covered with short yellowish-white hairs between middle and apical one-quarter...................................................................................***G. venata venata***
- Elytra densely covered with short yellowish-white hairs between middle and apical one-quarter...............................................................................***G. venata takakuwai***
3. Post-basal bumps of elytra covered with long black setae......................................................................................................................................................***G. obliquefasciata***
- Post-basal bumps of elytra covered with short black and long brown setae....................................................................................................................................***G. jinfoensis***

***Paratimiola* Breuning, 1965**

*Paratimiola* Breuning, 1965: 34 [7]; Rondon and Breuning 1970: 493 (catalogue) [34]; Breuning 1975: 7 (key) [29]; Breuning 1976: 137 (redescription) [30]. Type species: *Paratimiola rondoni* Breuning, 1965, by original designation.

By examining different types of *Tuberenes vietnamensis* Breuning, 1972, we found that it does not belong to *Tuberenes* because it lacks several diagnostic characteristics of this genus: each elytron is widely rounded at the apex, neither covered with a small fascicle of setae behind the base nor covered with white pubescence forming several longitudinal lines at the apical half, and the femora is not covered with erect pubescence.

**Figure 4:**
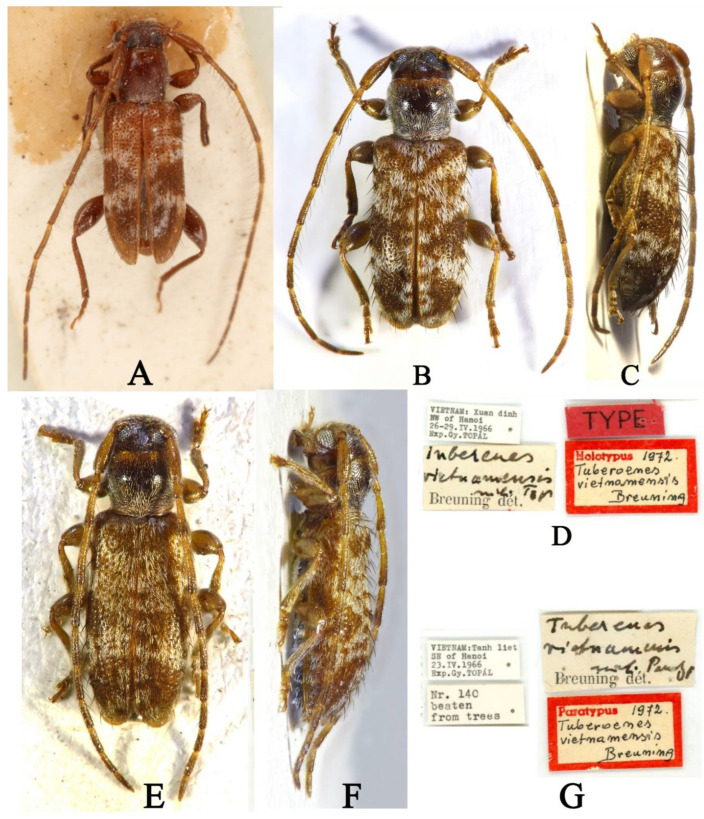
(**A**) *Paratimiola rondoni*, holotype, dorsal habitus (photograph (**A**) was taken by Junsuke Yamasako). (**B**–**G**) *Tuberoenes vietnamensis* types: (**B**–**D**) Holotype: (**B**) dorsal habitus; (**C**) lateral habitus; (**D**) labels. (**E**–**G**) Paratype: (**E**) dorsal habitus; (**F**) lateral habitus; (**G**) labels (photographs (**B**–**G**) were taken by Aranka Grabant).

Comparative analyses of the types of *Paratimiola rondoni* (Figure 4A) and *T*. *vietnamensis* (Figure 4B,C,E,F) reveal that both species obviously belong to same genus based on the shared commonality of morphological characteristics: antennae distinctly longer than the body; thick scape; pedicle longer than width; antennomere III slightly shorter than the scape; antennomere IV distinctly longer than antennomere III; antennomere V distinctly shorter than antennomere IV; length of antennomeres V–XI gradually reduced; pronotum with a pair of triangular tubercles at the middle sides; scutellum linguiform; elytra wider than the pronotum at the base; sides of elytra sub-paralleled at the basal third, slightly expanded in the middle, gradually constricted from the apical third to the apex and widely rounded at the apices; disc sparsely covered with erected long black setae, densely covered with short white pubescence at the basal third, forming irregular spots, and densely covered with short white pubescence in the middle, forming an X-shaped band, with coarse and moderately dense punctuations from the base to the apical third (the punctations are sparse from the apical third to the apex); the mesocoxal cavities open externally to the mesepimera; the femora of the legs are strongly clavate; and the mesotibiae have an outer groove in front of the apex.

*Paratimiola* currently consists of two species.

**Distribution:** Laos, Vietnam.

***Paratimiola rondoni* Breuning, 1965**

*Paratimiola rondoni* Breuning, 1965: 35 (type locality: “Phou Kow Khouei, près de Vientiane, Laos”) [7]; Rondon and Breuning 1970: 493 (catalogue), fig. 38 i [34]; Breuning 1976: 137 (redescription) [30].

**Type material examined:** Holotype (BPBM): Phou Kow Khouei region, a high-altitude station near Vientiane, 15 April 1965 (collecting data cited from Breuning 1965 [7]), examined from one photograph (Figure 4A).

**Diagnosis:** *Paratimiola rondoni* is very similar to *P*. *vietnamensis* (Breuning, 1972), as mentioned above, but can be clearly distinguished from *P. vietnamensis* by two diagnostic characteristics that are lacking in this species: the elytral base is not covered with short white pubescence, forming a V-shaped band surrounding the scutellum, and the apical third of each elytron is not covered with short white pubescence, forming a U-shaped band along the margin.

**Distribution:** Laos (Vientiane).

***Paratimiola vietnamensis* (Breuning, 1972) comb. nov.**

*Tuberoenes vietnamensis* Breuning, 1972: 237 (type locality: “Xuan dinh, NW of Hanoi, Vietnam”) [35].

*Tuberenes vietnamensis*: Breuning 1978: 21 (redescription) [8].

**Type material examined:** Holotype (HNHM): VIETNAM: Xuan dinh NW of Hanoi 26-29.IV.1966 Bxp. Gy. TOPÁL (printed with black ink on a rectangular white label)/*Tuberenes vietnamensis* mihi Typ Breuning dét. (“*Tuberenes vietnamensis* mihi Typ*”* handwritten and “Breuning dét.” printed with black ink on a rectangular white label)/TYPE (printed with black ink on a rectangular red label)/Holotypus 1972. *Tuberoenes vietnamensis* Breuning (“Holotypus” printed with red ink and “1972. *Tuberoenes vietnamensis* Breuning” handwritten with black ink on a rectangular white label with red borders), examined from three photographs (Figure 4B–D). Paratype (HNHM): VIETNAM: Tanh liet SE of Hanoi 23.IV.1966 Bxp. Gy. TOPÁL (printed with black ink on a rectangular white label)/Nr. 140 beaten from trees (printed with black ink on a rectangular white label)/*Tuberenes vietnamensis* mihi Paratyp Breuning dét. (“*Tuberenes vietnamensis* mihi Paratyp” handwritten and “Breuning dét.” printed with black ink on a rectangular white label)/Paratypus 1972. *Tuberoenes vietnamensis* Breuning (“Paratypus” printed with red ink and “1972. *Tuberoemes vietnamensis* Breuning” handwritten with black ink on a rectangular white label with red borders), examined from three photographs (Figure 4E–G).

**Distribution:** Vietnam (Hanoi).

***Tuberenes* Breuning, 1978**

*Tuberoenes* Breuning, 1972: 237 nomen nudum [35].

*Tuberenes* Breuning, 1978: 20 [8]; Löbl and Smetana 2010: 213 (catalogue) [10]; Lin and Yang 2019: 224 (catalogue) [27]; Danilevsky 2020: 299 (catalogue) [13]. Type species: *Eryssamena robustipes* Pic, 1939.

Breuning described *Tuberoenes* (sic) *vietnamensis* from Vietnam and compared it with *robustipes* Pic (this should be *Eryssamena robustipes* Pic, 1939, which is a type species of *Tuberenes*) [35], but there is no information on the genus *Tuberoenes*. Then, Breuning transferred *Tuberoenes vietnamensis* to *Tuberenes* when he first established *Tuberenes* [8]. While the Titan Database considers *Tuberoenes* a misspelling of *Tuberenes* [3], it is actually a nomen nudum following the ICZN, Art. 13.1.1 and Art. 13.3 [17].

**Distribution:** China, India.

***Tuberenes robustipes* (Pic, 1939)**

*Eryssamena robustipes* Pic, 1939: 15 (type locality: “Kansou, Chine = Gansu, China”) [36]; Gressitt 1951: 523 (catalogue) [37]; Breuning 1963: 524 (catalogue) [9]; Hua 2002: 207 (catalogue) [22].

*Tuberenes robustipes*: Breuning 1978: 20 (redescription) [8]; Löbl and Smetana 2010: 213 (catalogue) [10]; Mitra et al., 2017: 67 (fauna) [38]; Lin and Yang 2019: 224 (catalogue) [27]; Danilevsky 2020: 299 (catalogue) [13].

Since *Tuberenes minuta* (Pic, 1925) and *T*. *sikkimensis* Breuning, 1978 have been transferred to *Pararondibilis* and *T*. *vietnamensis* Breuning, 1972 has been transferred to *Paratimiola*, *Tuberenes* now consists of only one species.

**Figure 5:**
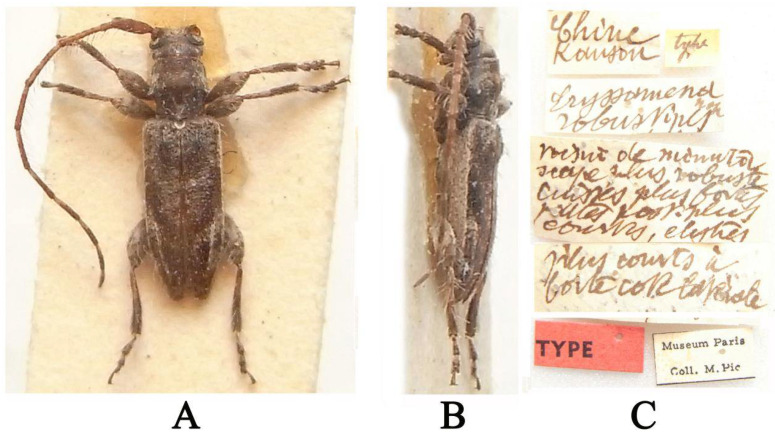
(**A**–**C**) *Eryssamena robustipes*, holotype: (**A**) dorsal habitus; (**B**) lateral habitus; (**C**) labels (photographs (**A**–**C**) were taken by Xavier Gouverneur).

**Type material examined:** Holotype (MNHN): Chine Kansou (handwritten with black ink on a rectangular white label)/type (handwritten with black ink on a rectangular yellow label)/*Eryssamena robustipes* n sp (handwritten with black ink on a rectangular white label)/voisin de *minuta* (i.e., *Eryssamena minuta* Pic, 1925), scape plus robuste, cuisses plus fortes, pattes post [érieures] plus courtes (the contents mean “similar to minuta, more robust scape, stronger thighs shorter hind legs” and were handwritten with black ink on a rectangular white label)/élytres plus courts à fortes côtes latérales (“elytres” was handwritten with black ink at the end of the previous label; the contents mean “shorter elytra with strong lateral ribs” and were handwritten with black ink on a rectangular white label)/TYPE (printed with black ink on a rectangular red label)/Museum Paris Coll. M. Pic (printed with black ink on a rectangular white label with black borders), examined from three photographs (Figure 5A–C).

**Distribution:** China (Gansu), India (Sikkim, Darjeeling).

## Data Availability

The original contributions presented in this study are included in the article. Further inquiries can be directed to the corresponding author.
